# Tensile Resistance and Fracture Mechanisms of Silica Aerogels Reinforced by Nanotube–Graphene Hybrid Networks

**DOI:** 10.3390/gels11060471

**Published:** 2025-06-19

**Authors:** Lin Guo, Mu Du, Jiaqian Li, Wei Li, Mingyang Yang, Gongming Xin

**Affiliations:** 1 Shenzhen Research Institute of Shandong University, Shenzhen 518000, China; lin.g@sdu.edu.cn (L.G.); dumu@sdu.edu.cn (M.D.); jiaqianli@sdu.edu.cn (J.L.); 2 School of Energy and Power Engineering, Shandong University, Jinan 250100, China; 3 Institute for Advanced Technology, Shandong University, Jinan 250100, China; 4 Interdisciplinary Research Center, School of Mechanical Engineering, Shanghai Jiao Tong University, Shanghai 200240, China; meweili@sjtu.edu.cn; 5 School of Resources Engineering, Xi’an University of Architecture and Technology, Xi’an 710055, China

**Keywords:** carbon-reinforced silica aerogel, tensile behavior, fracture mechanism

## Abstract

Despite their outstanding thermal insulation and ultralight structure, silica aerogels suffer from inherent mechanical fragility, making the investigation of their mechanical behavior crucial for expanding their practical utility in advanced applications. To enhance their mechanical performance, this study introduces a dual-phase reinforcement strategy by anisotropically incorporating carbon nanotubes (CNTs) and graphene oxide (GO) sheets into the aerogel matrix. Using molecular dynamic simulations, we systematically investigate the tensile behavior and pore structure evolution of these hetero-structured composites. The results reveal a non-monotonic dependence of tensile strength on loading ratio, distinguishing three strain-dependent reinforcement regimes. High loading content (11.1%) significantly improves strength under low strain (0–26%), whereas low loading levels (1.8%) are more effective at preserving structural integrity under large strain (44–50%). Moderate loading (5.1%) yields balanced performance in intermediate regimes. While increasing carbon content reduces initial pore size by partially filling the framework, tensile deformation leads to interfacial debonding and the formation of larger pores due to CNT–GO hybrid structure interactions. This work elucidates a dual reinforcement mechanism—physical pore confinement and interfacial coupling—highlighting the critical role of nanostructure geometry in tuning strain-specific mechanical responses. The findings provide mechanistic insights into anisotropic nanocomposite behavior and offer guidance for designing robust porous materials for structural and functional applications.

## 1. Introduction

Nanoporous materials have emerged as revolutionary candidates in energy storage and renewable energy systems owing to their unique multiscale structural characteristics [1]. Among these, aerogels represent a class of three-dimensional nanoporous solid materials whose characteristic architecture arises from the self-assembly of colloidal nanoparticles or polymer molecules through sol–gel processes [2], with gaseous phases occupying over 90% of their volume [3]. Dubbed “frozen smoke” for their record-low density (e.g., ~3 kg/m³ for silica aerogels) [4], these ultralight materials exhibit extraordinary physical properties, including nanoscale pore confinement effects and hierarchical porosity spanning from micro- to macro-dimensions [5].

Particularly noteworthy is silica aerogel, a non-crystalline nanoporous solid featuring an interconnected mesoporous network [6]. This material demonstrates exceptional characteristics, such as ultrahigh specific surface area (>800 m^2^/g) [7], tunable porosity (>95%) [8], and ultralow thermal conductivity (<0.02 W/m·K) [9], which underpin its versatile applications, ranging from spacecraft thermal protection [10] systems to novel frontiers in CO_2_ capture [5], flexible electronics [11], etc., establishing silica aerogels as a paradigm-shifting material platform in nanotechnology [12].

However, their widespread application is hindered by inherent mechanical fragility [13]. Silica aerogels exhibit a colloidal network of weakly connected “pearl necklace”-like nanoparticles [14], resulting in low elastic modulus (typically <1 MPa for native silica aerogels) and compressive strength (<0.1 MPa) [15]. Even optimized synthesis protocols yield materials prone to irreversible structural collapse at tensile strains exceeding 80%, despite their remarkable compressibility [16]. This brittleness stems from the nanoscale architecture-aging processes, which minimally enhance interparticle necking, while increased density through prolonged aging paradoxically induces glass-like fracture behavior [17]. NASA’s projection of aerogels as “21st-century plastics” [18] remains constrained by the fundamental trade-off between mechanical robustness and functional properties: strategies to improve strength, such as polymer cross-linking or fiber reinforcement [19], invariably elevate density and reduce porosity, compromising the ultralow thermal conductivity that defines their industrial value. Moreover, homogeneous networks derived from single-component precursors lack the hierarchical heterogeneity necessary to decouple mechanical reinforcement from pore structure degradation [20]. These limitations underscore the imperative for multicomponent hybridization and multiscale structural engineering to reconcile mechanical durability with functional performance in aerogel design [21].

To address these challenges, carbon-based nanomaterials have emerged as ideal candidates for hybridization due to their complementary mechanical and functional properties [22]. Structural hybridization stands as a pivotal strategy for enhancing the performance of three-dimensional porous network materials. Carbon-based nanoporous materials have attracted considerable attention due to their porous architecture, ultralow mass density, and exceptional mechanical properties [23]. The surface effects, quantum confinement, and dimensional characteristics inherent to these materials endow them with unique mechanical, thermal, optical, and electromagnetic functionalities, offering innovative solutions to energy and environmental challenges [24]. Graphene, with its sp^2^-hybridized bonding configuration, exhibits extraordinary mechanical properties, including a Young’s modulus of 1.1 TPa and intrinsic strength of 125 GPa-surpassing high-grade alloy steels by two orders of magnitude, and ranking among the strongest known materials [25]. Despite these merits, current research predominantly focuses on its optoelectronic applications, while its potential as a structural reinforcement phase remains underexploited.

Furthermore, carbon nanotubes (CNTs), as quasi-one-dimensional quantum materials, feature tubular walls composed of hexagonal carbon rings forming a honeycomb lattice, capped with fullerene-like hemispherical structures comprising alternating pentagonal and hexagonal carbon rings [26]. This unique geometry, combined with cost-effective synthesis, superior thermomechanical properties, and low density, positions CNTs as a focal point in nanocomposite research. When integrated into aerogel matrices, CNTs significantly enhance mechanical toughness and thermal stability [27]. Their nanoscale dimensions and high aspect ratios (>1000) facilitate uniform dispersion within aerogel networks, optimizing pore connectivity and specific surface area [28]. Therefore, by synergistically incorporating two-dimensional graphene and CNTs into silica aerogel frameworks, hybrid composites are expected to achieve not only reinforced mechanical performance but also hierarchical pore structures that enable exceptional adsorption site density and thermal insulation [29].

Despite these advances, the mechanical reinforcement mechanisms of carbon–silica hybrids remain to be explored. Macroscopic testing (e.g., universal testing machines) [30] and continuum-based simulations (e.g., finite element analysis) [31] can capture bulk mechanical responses. Still, the multiscale pore architecture (micropores < 2 nm, mesopores 2–50 nm, macropores > 50 nm) and complex deformation mechanisms (e.g., pore-wall buckling, particle sliding, and interface debonding) hinder precise resolution of microscale strain fields. Traditional constitutive models often rely on isotropic assumptions [32], which might not be applicable to porous reinforced composite materials. Characterization techniques, such as scanning electron microscopy (SEM) and transmission electron microscopy (TEM), despite achieving sub-nanometer spatial resolution, lack the temporal resolution to dynamically track lattice distortions (<0.1% strain sensitivity) or defect evolution (e.g., dislocation motion, crack nucleation) under mechanical loading. Experimental techniques, like in situ TEM or atomic force microscopy, despite achieving atomic spatial resolution, lack the temporal bandwidth to resolve sub-picosecond bond rupture events or progressive defect propagation under dynamic loading.

Molecular dynamics (MD) simulations have emerged as a powerful tool for probing the mechanical behavior of silica aerogels at the atomic level, offering unparalleled spatial and temporal resolution compared to traditional experimental techniques [33]. Unlike macroscopic tests, MD enable the direct observation of nanoscale deformation mechanisms such as ligament bending, shear localization, and crack initiation within the intricate three-dimensional network of aerogels [34]. By capturing the atomic interactions and dynamic responses under various loading conditions, MD simulations provide insights into how structural parameters, such as density, porosity, and the size and connectivity of silica nanoparticles, influence mechanical properties, like elastic modulus, tensile strength, and fracture behavior [35,36]. Furthermore, MD facilitate systematic exploration of reinforcement strategies, such as the inclusion of glass fibers, graphene sheets, or carbon nanotubes, allowing quantitative assessment of their toughening mechanisms and load-transfer efficiency [37,38]. This predictive capability is especially valuable for materials like aerogels, whose high porosity and structural heterogeneity present challenges for continuum modeling. Overall, MD simulations not only bridge the gap between nanoscale structure and macroscale behavior but also support the rational design of mechanically robust aerogel-based nanocomposites for advanced engineering applications. While previous computational studies, such as that of Patil et al. [36], have provided valuable insights into silica aerogels reinforced solely with CNTs, the mechanical behavior arising from the co-incorporation of anisotropically distributed one-dimensional CNTs and two-dimensional graphene oxide (GO) sheets remains less understood. The interplay between these distinct nanostructures, their potential synergistic or even antagonistic effects under mechanical loading, and how their combined presence influences strain-dependent failure mechanisms represent critical knowledge gaps.

Therefore, the present study employs MD simulations to construct silica aerogel models incorporating hybrid networks of anisotropically dispersed CNTs and GO sheets. Our primary objectives are as follows: (1) to systematically investigate the tensile behavior across a range of CNT–GO incorporation content and identify potentially strain-dependent reinforcement regimes specific to this hybrid system; (2) to elucidate the fracture mechanisms, paying particular attention to the role of CNT–GO interactions and the evolution of interfacial heterostructures; and (3) to analyze the dynamic changes in pore structure under tensile deformation as influenced by these hybrid reinforcements. This work aims to provide novel mechanistic insights into the complex behavior of such hybrid nanocomposites, offering guidance for designing robust porous materials with tailored, strain-specific mechanical responses that go beyond the understanding derived from single-component reinforced systems.

## 2. Results and Discussion

### 2.1. Resistance to Tension

**Figure 1:**
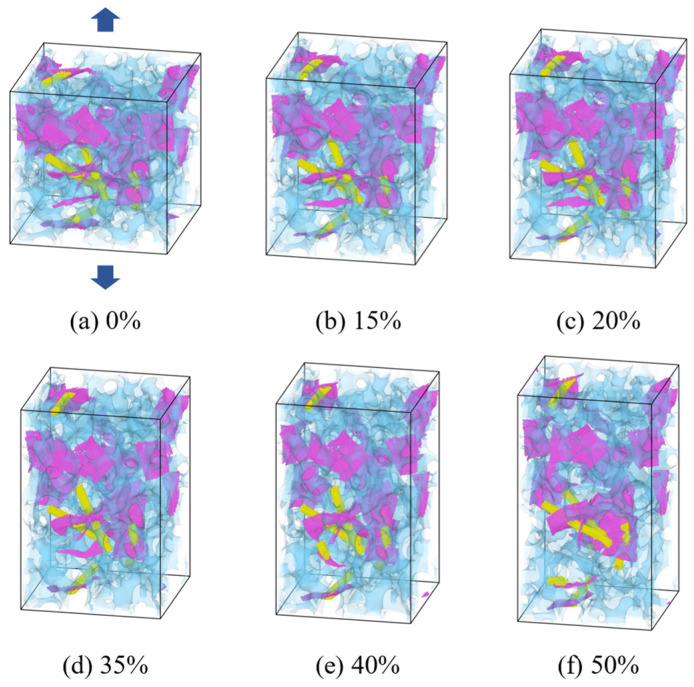
Deformation of composite aerogel during stretching process. (*w* = 1.8%).

Figure 1 illustrates the structural evolution of the aerogel under varying tensile strains, taking the case of a carbon nanotube (CNT) loading mass fraction of 1.8% as an example. As the tensile force increases, the aerogel monolith exhibits a progressive elongation in the longitudinal direction and a narrowing in the transverse direction, indicating substantial deformation. Additionally, Table 1 summarizes the dimensional changes of the computational domain during tensile loading for composite aerogels with different CNT loading levels. It is evident that the height increases under all conditions, confirming the occurrence of mechanical deformation across all tested samples.

**Figure 2:**
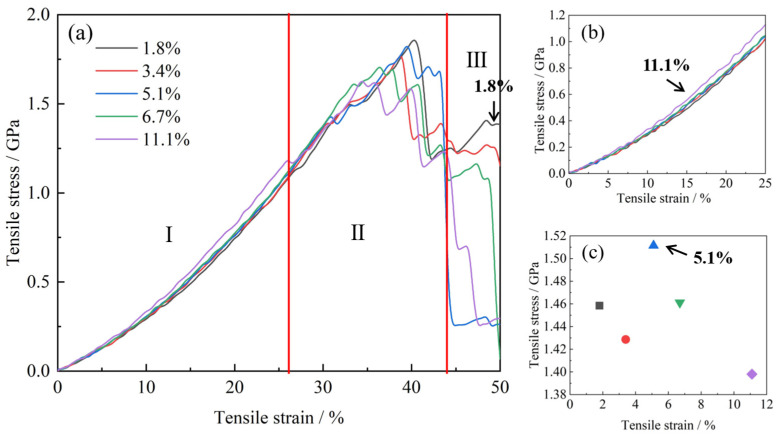
(**a**) Stress–strain curve under varying loading ratio, which can be divided into three modes: I (Tensile strain 0–26%), II (26–44%), III (44–50%). (**b**) Enlarged detail of Mode I, where the loaded rato of 11.1% has the best performance; (**c**) Averaged tensile stress of Mode II, where doped ration 5.1% has the best performance.

Figure 2 presents the stress–strain responses of aerogels with varying CNT loading ratios (1.8%, 3.4%, 5.1%, 6.7%, and 11.1%). Prior to further analysis, it is essential to validate the molecular dynamics (MD) simulation results. In our previous work [39], we have calculated the elasticity modulus (E) of pure silica aerogel (ρ = 0.48 g/cm^3^), which is 0.28 GPa. This value was verified by showing good agreement with values in the literature [40,41,42]. Patil et al. [36] demonstrate that reinforcing silica aerogels with CNTs (ranging from ~2 wt% to ~10 wt%) significantly enhances tensile strength, with reported values typically falling in the range of approximately 0–1.8 GPa. This represents a substantial improvement over pure silica aerogels, which exhibit much lower strengths. In our study of CNT–GO hybrid reinforced silica aerogels (Figure 2a), the simulated tensile strengths for the composites are observed in the range of approximately 0–1.8 GPa. This consistency in the order of magnitude of tensile strength reinforced the validity of the present model.

As shown in Figure 2a, the tensile strength of the material exhibits a non-monotonic dependence on CNT loading ratio, initially increasing and then decreasing. The stress-strain behavior can be broadly classified into three regimes. (1) Low-strain regime (<26% strain) Moderate CNT loading enhances tensile performance. Notably, the sample with 11.1% CNTs shows a pronounced improvement in tensile strength, whereas the other loading levels result in only marginal gains (Figure 2b). (2) Intermediate-strain regime (26–44% strain): While tensile strength generally increases with reinforcement contents, this correlation becomes less consistent at lower loading levels, suggesting a reduced sensitivity of mechanical response to CNT content in this strain range. To account for the fluctuations, we analyzed the average stress within this regime and found that the sample incorporated with 5.1% CNTs demonstrates the highest tensile performance (Figure 2c). (3) High-strain regime (44–50% strain): The mechanical behavior undergoes a significant reversal. As seen in Figure 2a (Region III), the low-loaded sample (1.8%) exhibits the best tensile performance, maintaining a high stress level even at 50% strain, indicating robust structural integrity.

Importantly, a sudden drop in tensile strength is observed for all samples near the critical strain of 44%. The 5.1% CNT-reinforced aerogel displays relatively superior performance, with fracture delayed to 43% strain. However, unlike other samples, it does not show a stepwise stress decay. Based on these observations, the tensile response is categorized into three typical regimes: (1) 0–26% strain: optimal performance with 11.1% CNTs; (2) 26–44% strain: highest strength with 5.1% CNTs; (3) 44–50% strain: 1.8% CNT-reinforced sample shows the best mechanical integrity. These findings reveal a distinct strain-dependent reinforcement mechanism of CNTs: at moderate deformation, CNTs effectively preserve the pore structure and enhance mechanical stability, whereas excessive strain is likely to lead to interfacial debonding between the CNTs and the matrix, reducing the reinforcing effect.

Previous studies on silica aerogels reinforced solely with carbon nanotubes have generally reported a monotonic enhancement in mechanical properties with increasing CNT content. For instance, Patil et al. [36] demonstrated that even a small addition of double-walled CNTs (DWCNTs) (e.g., 2.06 wt%) led to a significant increase in tensile strength and elastic modulus, attributed to the effective load-bearing capacity of the well-dispersed, strong, and stiff DWCNT fibers. Studies focusing on GO-only reinforced silica aerogels highlight different aspects. Our co-authored work [39] revealed that graphene sheets, when incorporated into silica aerogels, provided limited direct enhancement to tensile strength. At higher strains, graphene sheets were observed to slip from the silica matrix due to the relatively weak, non-covalent graphene-silica interactions, especially for non-functionalized sheets. In contrast, our hybrid CNT–GO system exhibits a more complex, non-monotonic relationship between total carbon reinforcement and tensile performance, particularly in different strain regimes. To unravel the specific structural and interfacial mechanisms responsible for this behavior, we undertook a series of more detailed investigations.

### 2.2. Fracture Behavior

**Figure 3:**
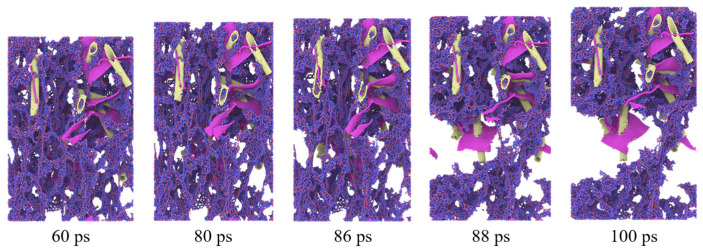
Tensile fracture of the composite aerogel (*w* = 5.1%).

During the tensile process, progressive deformation and failure of the carbon-based reinforcements were observed, as illustrated in Figure 3. These carbon nanostructures, initially serving as critical load-bearing elements within the aerogel framework, began to exhibit fatigue-like characteristics under sustained tensile loading. Fracture initiated at the central region whereas, in others, distinct crack paths developed along specific two-dimensional planes. This failure process was gradual in nature and its severity showed a clear positive correlation with the applied tensile strain.

**Figure 4:**
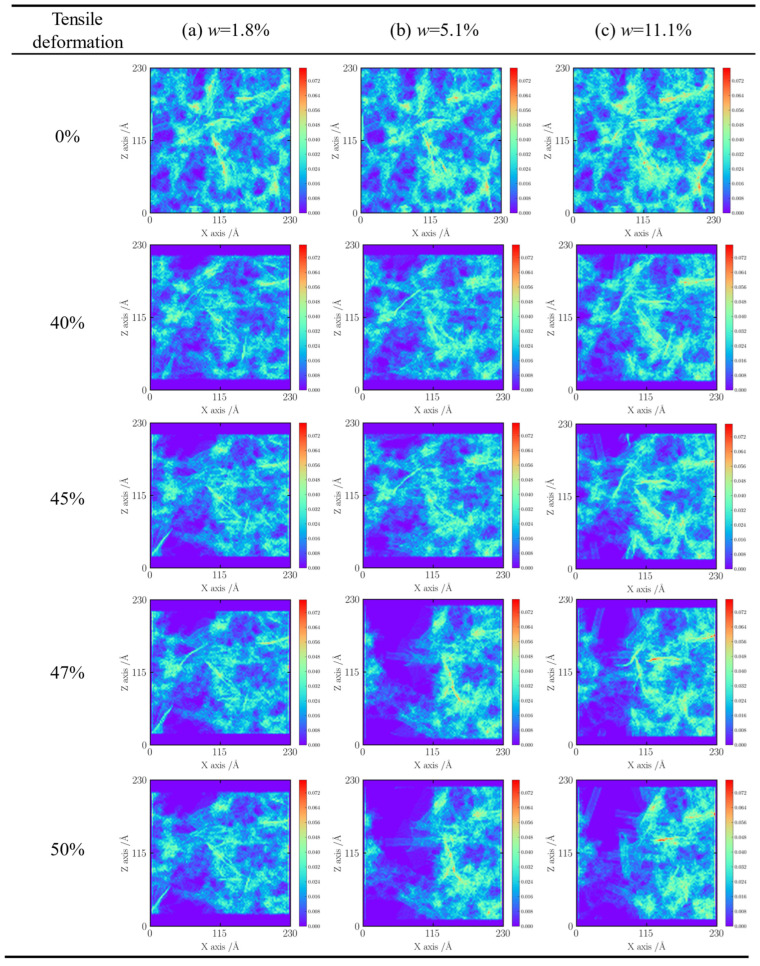
Material density contours.

Density mapping of the silica aerogel/carbon nanotube (CNT) composite with a loading mass fraction of 1.8% (Figure 4a) reveals a noticeable asymmetry in pore distribution; more pronounced porosity appears on the left side of the sample’s central region compared to the right. The red and yellow regions in the density plot correspond to zones of elevated atomic density, potentially indicating localized microstructural heterogeneity or abrupt changes in physical properties. These high-density regions are presumed to be composed predominantly of interwoven graphene sheets and CNT networks, which exhibit superior mechanical stability under tensile loading, with only localized failure observed.

Comparative analysis across samples with varying CNT content suggests a consistent pattern in the distribution of these high-density domains, lending further support to the hypothesis of microstructural regularity in these regions. Notably, within the low-strain regime (0–16%), the pore structure undergoes oriented elongation, leading to a systematic decrease in material density, as reflected by the overall lightening of the density map. At a strain of 33.5%, the pore network extends to the sample boundaries, and the trend of density reduction becomes more pronounced. The fading color in the contour maps is indicative of internal stress redistribution: as the external load increases, localized stress concentrations surpass the material’s load-bearing threshold, initiating fracture. The marked fading at the sample edges serves as a visual representation of microcrack nucleation.

For the sample with 5.1% CNT loading ratio (Figure 4b), the density map reveals a significant increase in the number of distinct microstructural features, as indicated by the red-circled regions and irregularly shaped, high-brightness, high-density zones. These heterostructures exacerbate tearing behavior during the later stages of deformation. Although the evolution of porosity within the 0–33.5% strain range is qualitatively similar to that of the lower-loaded sample, the degree of color fading is notably greater, suggesting more intense pore structure reconfiguration. The circled high-density zones were confirmed to be CNT-graphene heterostructures with non-classical geometries-neither purely tubular nor sheet-like, likely formed through structural rearrangement during the fabrication process.

**Figure 5:**
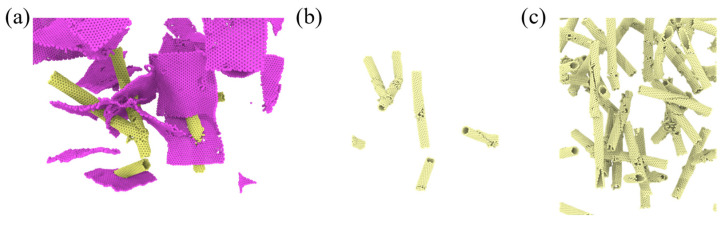
Details of the fractured nanotubes (**a**–**c**).

A comprehensive analysis of multiple simulation datasets reveals a consistent trend: increasing the CNT reinforcement content promotes the formation of special microstructures within the aerogel matrix (Figure 5a). These structures facilitate pore proliferation and enlargement during tensile loading, ultimately compromising the tensile performance of the composite. Systematic comparisons of density maps across various strain levels further indicate a coordinated evolution between the pore network and these microstructural features. Such strain-induced reconfiguration at the microscale provides critical insight into the underlying mechanisms governing macroscopic failure.

During the deformation process, both CNTs and graphene sheets largely maintain structural integrity, with only a minority exhibiting visible fracture. However, as the CNT incorporation level increases, the frequency of CNT breakage rises markedly. At a CNT mass fraction of 11.1%, the number of fractured CNTs after tensile loading is significantly higher than in lower-loaded samples. As shown in Figure 5b, the observed fracture morphologies of CNTs display diverse patterns, including the following: (i) individual CNTs breaking at mid-length with various orientations, (ii) interwoven CNTs fracturing at intersection points, and (iii) irregular, non-patterned breakage scenarios.

Notably, CNT incorporation has minimal impact on tensile strength when the strain remains below 44%. However, in the high-strain regime (44–50%), increasing the CNT content correlates with a noticeable decline in mechanical strength. This deterioration is attributed to the coupled interaction between CNTs and graphene sheets, wherein mutual interference under tensile stress reduces the stability of the composite framework. Given the relatively fragile nature of the CNT–graphene hybrid structure, this interaction leads to premature failure and degrades the overall tensile performance of the material.

This discrepancy suggests a complex, and at times antagonistic, interaction between graphene and CNTs under deformation. Firstly, the interface between the 1D CNTs and the 2D GO sheets, particularly at their junctions or edges, may act as stress concentration sites, especially if there is an abrupt change in stiffness or geometry. This could lead to premature localized failure under tensile load. Secondly, the relatively weak van der Waals forces governing the adhesion at GO–silica and GO–CNT interfaces might be insufficient to prevent interfacial debonding, especially if local structural disorder or unfavorable orientations of GO sheets (e.g., perpendicular to the principal stress direction) exist. Such debonding would create micro-voids and weaken the composite. Thirdly, the rigid, planar nature of GO sheets might impose geometric constraints on the deformation and rearrangement of the surrounding flexible silica network or CNTs. This could hinder effective stress dissipation mechanisms that are crucial for toughness, leading to strain localization and earlier fracture in regions influenced by GO.

**Figure 6:**
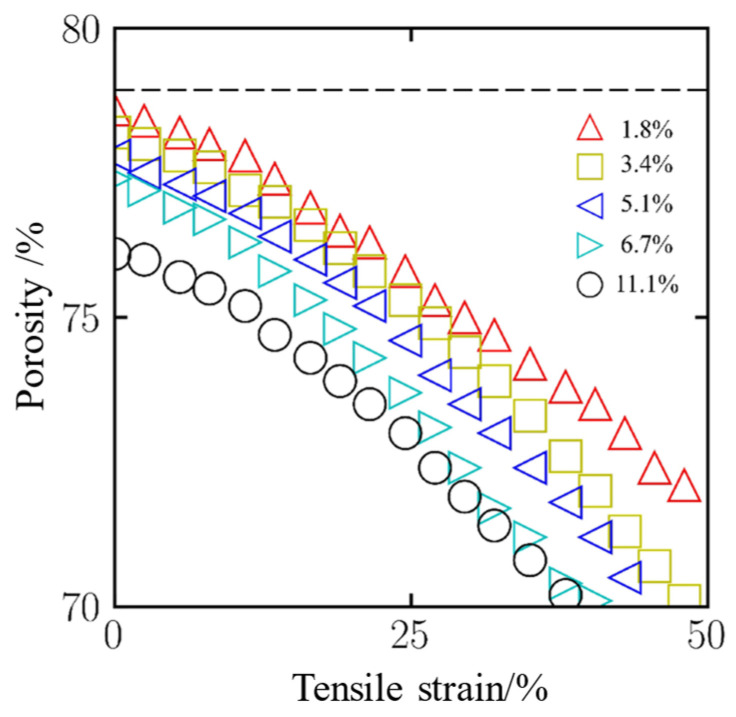
Porosity changes during tensile tress.

These hypothesized mechanisms, supported by the observed fracture patterns (e.g., Figure 5 and Figure 6, showing crack paths potentially influenced by these heterostructures), highlight the necessity for deeper investigation into these specific interfacial phenomena in hybrid systems. These observations are consistent with the hypothesized mechanisms where CNT–GO heterostructures can act as critical points. For instance, the irregularly shaped, high-brightness, high-density zones (Figure 4b) may represent regions where GO sheets create significant local stress fields or impede uniform load transfer, aligning with the concept of stress concentration at 1D–2D junctions. Similarly, the varied fracture morphologies of CNTs (Figure 5b), especially those interwoven CNTs fracturing at intersection points possibly involving GO, could be indicative of premature failure due to complex stress states or weakened interfaces in these hybrid regions rather than intrinsic CNT failure alone.

### 2.3. Pore Evolution

Porosity is a critical parameter that characterizes the microstructural features of aerogels, and its variation strongly influences both mechanical properties and functional performance. Analyzing the dynamic evolution of porosity during tensile deformation provides important insights into the role of CNT in stabilizing the pore architecture of aerogels, thereby offering a theoretical foundation for the structural optimization of these materials. As shown in Figure 6, the evolution of porosity with respect to engineering strain is quantitatively presented for aerogel composites with different CNT loading levels. The x-axis represents the tensile strain, while the y-axis denotes the relative change in porosity. The results indicate that increasing the CNT concentration leads to a gradual reduction in overall porosity. Concurrently, as the tensile strain increases, the structural porosity continuously decreases, suggesting that mechanical deformation progressively compresses and reorganizes the internal pore network.

**Figure 7:**
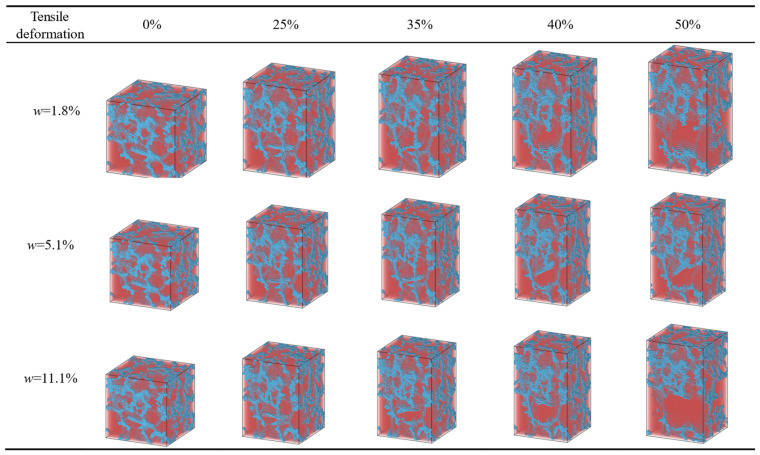
The evolution process of the pore structure of aerogel. The blue area represents the aerogel matrix, and the red area represents the pore structure.

The three-dimensional visualization capabilities of the OVITO 3.11.2 enable clear observation of the pore structure evolution in aerogels during tensile deformation. Figure 7 presents a sequence of snapshots illustrating the pore morphology of silica aerogels incorporated with CNTs at mass fractions of 1.8%, 5.1%, and 11.1%, under varying tensile strain levels (0%, 25%, 35%, 40%, and 50%). In these images, blue regions represent the aerogel matrix, while red regions indicate pore spaces.

The image sequences clearly reveal a progressive expansion of pore volume as the applied strain increases, with the formation of larger pore domains within the aerogel structure. When the strain reaches the critical range of 44–50%, local structural instabilities lead to the emergence of macroscopic voids. Comparative analysis further indicates that samples with higher CNT loading ratio exhibit more pronounced pore expansion than those with lower loading levels, such as the 1.8% sample. This suggests that CNT concentration plays a significant role in regulating deformation-induced pore evolution (Figure 7).

**Figure 8:**
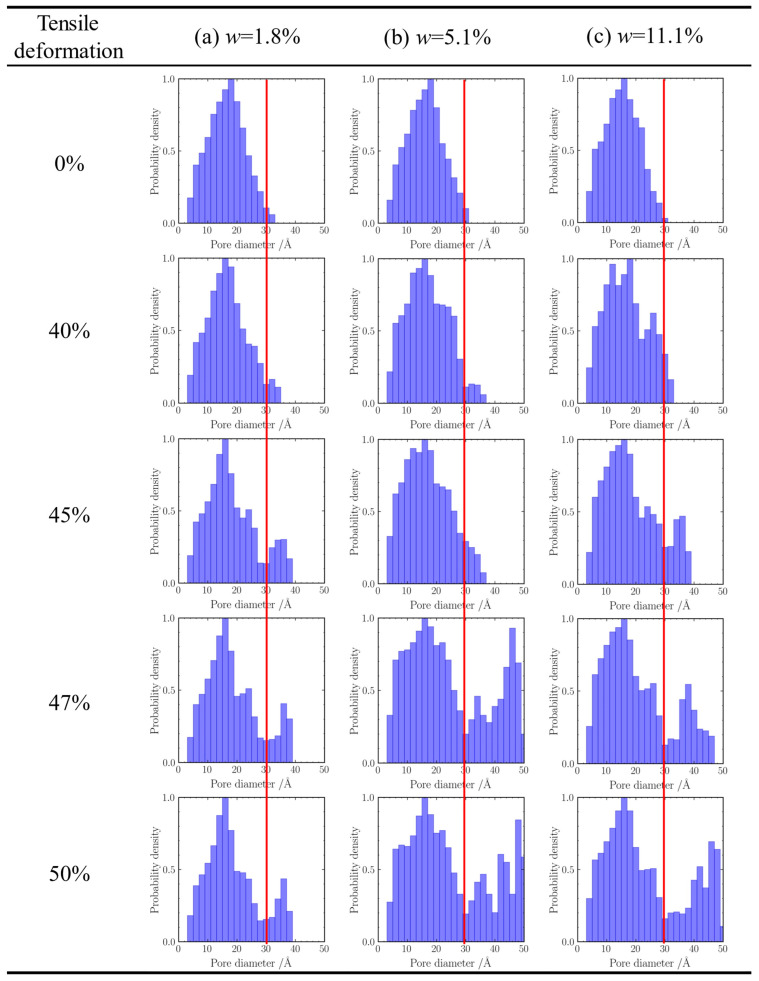
Pore size distribution of composite aerogel during tensile process.

In addition to visualizing pore morphology, we statistically analyzed the pore size distributions of the CNT-reinforced silica aerogel samples, as shown in Figure 8. Figure 8a presents the pore size histogram of the sample with a CNT mass fraction of 1.8%. Initially, the dominant pore size is 18 Å, with a pronounced probability density peak, and only a few pores exceeding 30 Å. As tensile strain increases, the peak probability gradually shifts toward smaller pore sizes and stabilizes at 16 Å. The probability densities across all pore size intervals exhibit an initial increase followed by a decrease. Specifically, the occurrence probabilities of 18 Å, 20 Å, and 22 Å pores drop significantly after deformation, whereas the 30–38 Å range, especially at 36 Å, shows a marked increase, with the maximum pore size expanding to 38 Å.

Figure 8b corresponds to the sample with a CNT mass fraction of 5.1%. Its initial distribution is also centered at 18 Å, with only trace amounts of 30 Å pores. Upon stretching, the peak shifts continuously to 16 Å. The overall probability density increases across the entire distribution, though slight decreases are observed at 18 Å and 20 Å. Notably, 48 Å emerges as the dominant large pore size, and the maximum pore size extends to 50 Å. Figure 8c shows the evolution in the 11.1% CNT-reinforced sample, where the 16 Å pores are initially dominant, with minor presence at 30 Å. During deformation, the probability peak exhibits oscillatory shifts but ultimately returns to 16 Å. Apart from slight reductions in 12 Å, 14 Å, 20 Å, and 22 Å pore probabilities, all other intervals show substantial increases. Among them, 46 Å becomes the most prevalent pore size in the >30 Å range, with the maximum pore size again reaching 50 Å.

Collectively, these results reveal two key trends. First, as CNT incorporation increases, the initial characteristic pore size decreases systematically, reflected by a leftward shift in the histogram peak from 18 Å to 16 Å. Second, post-tensile deformation leads to a rightward shift in the pore size distribution, with a growing prevalence of larger pores (>30 Å) at higher loading levels. The maximum pore sizes observed for the 1.8%, 5.1%, and 11.1% CNT-reinforced samples are 38 Å, 50 Å, and 50 Å, respectively.

Aerogels with different CNT loading levels exhibit distinct porosity evolution behaviors during tensile deformation. Under tensile stress, the microporous structure of the aerogel undergoes axial elongation, and adjacent pores may become interconnected, forming continuous mesoporous channels that contribute to a gradual increase in overall porosity. As the CNT loading level increases, the amplitude of porosity variation decreases progressively. Low-loaded systems (mass fraction ≤ 3.4%) show the least porosity fluctuation, confirming that the embedded CNT network enhances the mechanical stability of the aerogel and effectively suppresses the destabilization and coalescence of the pore structure.

Beyond the mechanical behavior, the observed evolution of the pore structure has significant implications for the functional performance of these aerogels. The rightward shift in pore size distribution, indicating an increase in the population of larger pores, could influence both thermal and mass transport properties. On the one hand, this may have a nuanced effect on the material’s thermal insulation capability. The exceptional insulating performance of silica aerogels relies heavily on the Knudsen effect, where small pore sizes (typically < 70 nm) suppress the gaseous contribution to thermal conductivity. The formation of larger pores, as seen in our simulations, might weaken this effect, potentially leading to a slight increase in thermal conductivity. On the other hand, for applications where mass transport is critical, such as catalysis, sensing, or filtration, the observed structural changes could be highly beneficial. A network of larger, interconnected pores would reduce the tortuosity and lower the resistance to fluid or gas flow, thereby enhancing diffusion and convection rates. This suggests a potential trade-off: while optimizing maximum tensile strength with high CNT loading might slightly compromise thermal insulation, it could concurrently improve the material’s efficiency as a high-performance filter or catalyst support. This highlights the multi-objective nature of designing functional aerogel composites, where the optimal reinforcement strategy depends heavily on the target application.

### 2.4. Discussion

A particularly noteworthy observation from Figure 2 is the non-monotonic relationship between the CNT reinforcement content and the tensile strength of the hybrid aerogels, especially when considered alongside the trend in decreasing initial porosity with increasing CNT content (as indicated by Figure 8 showing smaller initial characteristic pore sizes at higher CNT levels, e.g., 16 Å for 11.1% CNT vs. 18 Å for 1.8% CNT). While a reduction in porosity typically correlates with enhanced mechanical properties due to a more densified structure, our results show that, beyond a certain reinforcement level (e.g., 5.1% CNT content as optimal for the intermediate strain regime, and strength declining for 11.1% CNT content in the high strain regime compared to 1.8%), the tensile performance deteriorates. This counterintuitive behavior suggests that simply increasing the volume fraction of reinforcement, thereby reducing porosity, does not indefinitely improve strength and can indeed become detrimental.

Impaired load transfer efficiency can arise at high CNT concentrations. While individual CNTs are excellent load bearers, effective reinforcement relies on efficient stress transfer from the silica matrix to the CNTs and potentially between CNTs. At higher contents, the probability of CNT agglomeration or bundling increases. These agglomerates may behave as larger, less effective reinforcing units with a reduced effective interfacial area for stress transfer compared to well-dispersed individual CNTs and can also act as stress concentration points [36,43]. Increased CNT–CNT contacts may not always contribute positively if the inter-tube friction or bonding is weaker than the CNT–matrix interface, leading to slippage or premature failure at these junctions. The presence of GO sheets can further complicate load paths, potentially shielding CNTs from the matrix or creating weak CNT–GO interfacial regions that fail before the full load-bearing capacity of the CNTs is utilized, as hinted by our observations of CNT–GO hybrid structure interactions (Figure 5).

Excessive reinforcement can lead to a reduction in the composite network’s ability to accommodate deformation, effectively decreasing its flexibility and potentially increasing brittleness. The inherent silica aerogel network possesses some capacity for local rearrangement (e.g., bending of ligaments, reorientation of particles) to dissipate stress. However, a very dense network of rigid CNTs and GO sheets can significantly over-constrain the silica matrix, limiting these crucial local deformation mechanisms. This can transform the failure mode from a more distributed damage process to a more localized, brittle fracture at lower overall strains. The overall composite becomes much stiffer, which, if not accompanied by a proportional increase in toughness, can mean that less energy is required to initiate and propagate a crack. The significantly increased total interfacial area at high reinforcement content, especially the complex and potentially less optimized CNT–GO interfaces, introduces a higher density of potential defect sites or stress risers, which can become dominant failure initiation points, outweighing the benefits of a higher volume fraction of strong reinforcements.

Therefore, the observed decline in tensile strength at the highest CNT content (11.1% in certain strain regimes), despite reduced initial porosity, can be understood as a consequence of these detrimental effects on load transfer and network deformability becoming dominant over the benefits of simply adding more reinforcing material. This highlights a critical optimization challenge in designing such multi-component hybrid nanocomposites.

Despite the inherent nanoscale limitations, our MD findings offer significant scale-up implications for the rational design of macroscopic aerogel composites. The observed stress concentrations at CNT–GO junctions, for instance, highlight potential nanoscale weak points that could dictate bulk failure if not addressed through targeted interface engineering during synthesis. Similarly, the identified strain-dependent reinforcement regimes suggest that optimal reinforcement strategies for real-world applications may need to be tailored to the anticipated operational strain environments, moving beyond a one-size-fits-all approach. By revealing the incipient mechanisms of failure, such as the role of specific heterostructures in promoting tearing or the sequence of bond breakage, these simulations provide a foundational understanding that can guide the development of hierarchical architectures designed to arrest crack propagation at larger scales.

Furthermore, the potential for chemical bonding between functionalized CNTs (e.g., those bearing -COOH or -OH groups) and the silica matrix via condensation reactions (forming Si-O-C linkages) during the wet sol–gel synthesis is a significant factor not explicitly modeled in our current non-reactive MD simulations. Investigating these reactive bonding scenarios using advanced reactive force fields represents a valuable and complex direction for future computational work, which would more closely bridge the gap with experimentally synthesized materials. It is also important to note that the present study focuses exclusively on the uniaxial tensile response of the hybrid aerogels. While this provides fundamental insights into their fracture mechanics, real-world applications involve more complex loading conditions, such as compression, shear, and cyclic fatigue. A comprehensive investigation into the compressive and fatigue behavior of these CNT–GO hybrid systems represents a critical and promising avenue for future work.

## 3. Conclusions

In this study, molecular dynamics simulations were employed to investigate the tensile behavior of silica aerogels reinforced with anisotropically distributed carbon nanotubes (CNTs) and graphene oxide (GO) sheets at varying mass fractions. The analysis reveals the strain-dependent mechanical response and pore evolution of carbon-reinforced aerogels, underscoring the dual role of CNTs and GO in reinforcing the matrix while also potentially inducing failure due to interfacial instabilities at high loading levels. Unlike compressive stress responses, the tensile behavior exhibits a non-monotonic relationship between loading concentration and tensile strength, which can be categorized into three distinct strain-dependent performance regimes. As the loading level increases, the initial characteristic pore size decreases due to partial filling of the aerogel framework by carbon nanostructures. However, under tensile loading, the formation of CNT–GO hybrid structures leads to local interfacial debonding and fracture, which weakens the composite and facilitates the development of larger pores. A high loading ratio (11.1 wt%) is recommended for applications within the low-strain regime (0–26%), whereas a low loading ratio (1.8 wt%) is preferable in the high-strain regime (44–50%). These findings offer novel insights into anisotropic reinforcement mechanisms in porous nanocomposites and provide valuable guidance for the rational design of mechanically robust aerogel materials.

## 4. Method

### 4.1. Description of the Molecular Dynamics Simulation

Molecular dynamics (MD) simulations of silica aerogels were conducted using the Large-scale Atomic/Molecular Massively Parallel Simulator (LAMMPS) package. Silica aerogels are characterized by their highly complex and disordered structures, where atoms are arranged in a non-crystalline, random configuration. For models of the same bulk density, variations primarily arise from the spatial arrangement and connectivity of gel particles. In this study, we focus on a silica aerogel system incorporated with carbon nanotubes (CNTs) and graphene sheets. A fully atomistic structural model was constructed to investigate the mechanical behavior and pore evolution of the composite aerogel through MD simulations. The interatomic interactions within the silica network were described using the Vashishta potential, which accounts for both pairwise and angular (three-body) interactions. This potential captures not only the direct interactions between two atoms as a function of their separation distance but also incorporates energy terms associated with bond angles and the relative orientation of atom triplets. Consequently, it enables accurate reproduction of the amorphous structure and physical properties of SiO_2_. Mathematically, the total interaction energy is expressed as follows:

(1) $$V_{ij}=\frac{H_{ij}}{r^{\eta_{ij}}}+\frac{Z_{i}Z_{j}}{r_{ij}}exp(\frac{-r_{ij}}{r_{1s}})-\frac{P_{ij}}{r_{ij}^{4}}exp(\frac{-r_{ij}}{r_{4s}})$$

(2) $$V_{ijk}=B_{ijk}f(V_{ij}\tau_{ik})(cos\theta_{ijk}-cos{\bar{\theta }}_{ijk}{)}^{2}$$

Here, *r_ij_* is the distance between the *i*_th_ and *j*_th_ atoms, while *V_ij_* and *V_ijk_* represent the two-body and three-body interaction potentials, respectively. The first term on the right-hand side of Equation (1) corresponds to short-range repulsion, with *H_ij_* and *η_ij_* denoting the strength and exponent of the repulsive interaction. The second term represents the Coulombic interaction, where *Z_i_* is the effective charge on atom *i*, and *P_ij_* reflects the polarizability between atoms. *r*_1*s*_ and *r*_4*s*_ define the cutoff distances for interactions. In Equation (2), *B_jik_* specifies the strength of the three-body interaction, and *θ_jik_* denotes the bond angle between the vectors *r_ij_* and *r_ik_*. The complete form of the potential, along with the detailed parameter values, is available in the original work by Vashishta et al. [44,45].

### 4.2. Construction of Silica Aerogel Models with Varying Doping Concentrations

**Figure 9:**
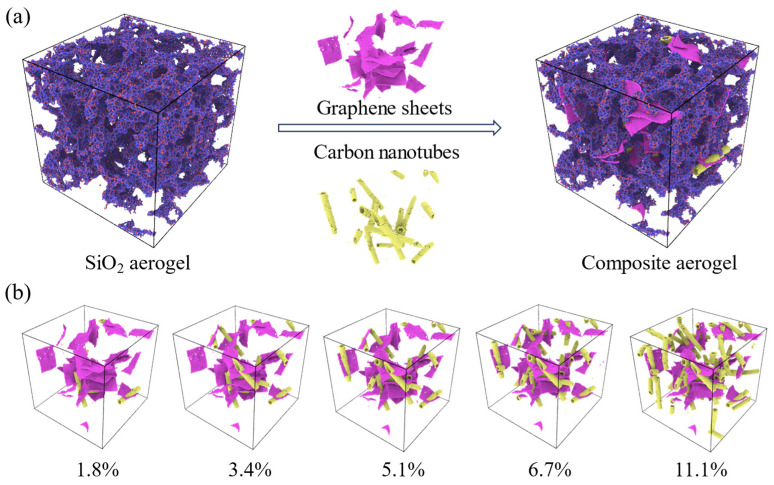
(**a**) Structure of the composite aerogel, which is randomly incorporated with graphene sheets and carbon nanotubes in SiO_2_ aerogel; (**b**) Anisotropic randomly reinforced structures with varying loaded ratio.

The pure silica aerogel (0.48 g/cm^3^, specific area of 1091 g/m^3^, averaged pore size of 1.7 nm) of 230 × 230 × 230 Å^3^ is generated by ‘‘negative rupturing’’ method and shown as Figure 9a. The details can be found in refs. [39,46,47]. Periodic boundary conditions were applied in all three dimensions to mimic an infinitely repeating system and minimize surface artifacts. The simulated volume might not capture the full statistical ensemble of all possible microstructural heterogeneities or rare defects that could be present in a macroscopic sample and potentially dictate its bulk failure. Nevertheless, the chosen system size allows for the detailed observation of fundamental failure initiation events at the nanoscale. MD simulations at this scale are powerful for elucidating fundamental nanoscale mechanisms and comparative trends.

While functionalization of CNTs is a common and effective strategy in experimental settings to improve dispersion and interfacial adhesion with the silica matrix [48], employing non-functionalized CNTs here serves to establish a baseline understanding. This approach helps isolate the effects of reinforcement content and distribution, providing a foundational reference for future studies that may explore the impact of specific CNT functionalization. In this study, non-functionalized CNTs were utilized. The all-atom graphene sheets (8 × 5 nm^2^) and carbon nanotubes are loaded into the pure silica aerogel with the same random seed to guarantee their distribution is highly random and homogeneous. The weigh percentage *w* is defined as a ratio of incorporated nanotubes mass to the mass of pure silica aerogel. The composite aerogels with varying doping ratios (*w* = 1.8%, 3.4%, 5.1%, 6.7%, 11.1%) are generated. Carbon atoms numbers to construct nanotubes and sheets are listed in Table 2 and their structures are shown as Figure 9b. To minimize the residual stress due to the loaded graphene, the system is firstly coupled with NVT ensemble at 300 K for 1 ns until the system energy reaching the lowest. The tensile simulations were conducted at a strain rate of 0.0004 ps^−^^1^. According to the study by Patil et al. [36], this strain rate for MD simulations is well-justified, a conclusion supported by a dedicated strain rate sensitivity analysis. Such strain rates and time scale are necessary to observe events within computationally feasible timescales and are widely accepted for studying fundamental deformation mechanisms and comparative trends in nanomaterials. The pore size of the aerogel model is defined as the largest particle that can pass through without overlapping with neighboring silica atoms. The details can be found in ref. [49].

## Figures and Tables

**Table 1:** Changes in computational box during stretching process.

Loading ratio	0%	1.8%	3.4%	5.1%	6.7%	11.1%
Length	232	187	189	205	193	204
Width	232	198	200	214	205	216
Height	232	343	343	344	344	347

**Table 2:** Numbers of loaded atoms under different doping ratios.

Loaded Ratio *w*	0%	1.8%	3.4%	5.1%	6.7%	11.1%
Carbon atom of nanotubes	0	6300	12,600	18,900	25,200	44,100
Carbon atom of graphene sheets	0	32,000	32,000	32,000	32,000	32,000

## Data Availability

The original contributions presented in this study are included in the article. Further inquiries can be directed to the corresponding authors.
